# Digital transformation and organizational restlessness

**DOI:** 10.3389/fsoc.2024.1430384

**Published:** 2024-09-10

**Authors:** Cristina Besio, Marco Jöstingmeier, Christine Posner

**Affiliations:** Chair for Organizational Sociology, Institute for Social Science, Helmut-Schmidt-University Hamburg, Hamburg, Germany

**Keywords:** digitalization, organizational restlessness, organizational change, innovation, public administration

## Abstract

In modern society, organizations are expected to be increasingly flexible and adapt to constantly changing environments. While such flexibility is often considered a positive trait of organizations, the risks of continuous organizational change are often overlooked. Against this background, we argue that continuous, multiple and uncoordinated organizational change can lead to a state we define as “organizational restlessness” and a loss of the benefits of stable structures. Paradoxically, it is even possible that organizational restlessness reduces the capability of organizations to planfully introduce specific and highly desirable changes, such as those related to digital transformation. Using qualitative data from interviews and participant observations, we analyze a large German public administration and identify three sources of organizational restlessness: the innovation imperative of modern society, changes in political leadership as a result of democratic elections and the bureaucratic principle of personnel rotation. While barriers to digital transformation are often explained by bureaucratic rigidity, we show that also constant uncoordinated change hinders sustainable digital transformation. Our paper thus contributes to an enhanced understanding of organizational continuity and disruption, as we show that both are needed to digitalize organizations further.

## 1 Introduction

In a society characterized by increasing complexity, rapid change and enduring crises, organizations are expected to be increasingly flexible and continuously learn and adapt to turbulent environments and their heterogeneous demands (*cf.* Gherardi, 2001; Nonaka and von Krogh, 2009; Argote, 2013; Ansell et al., 2017; Moreira, 2017; North et al., 2018; Örtenblad, 2020; Zaccaro et al., 2023). While flexibility is often considered a positive trait of organizations, we demonstrate that continuous, multiple and uncoordinated organizational changes can lead to a state we define as “organizational restlessness” and a loss of the benefits of stable structures. Paradoxically, it is even possible that organizational restlessness reduces the capability of organizations to introduce specific and highly desirable changes.

As a case study, we analyze how a large German administrative organization, the Federal Ministry of Defence [Bundesministerium der Verteidigung (BMVg)], deals with the current societal request to digitalize. It is common knowledge that this organization introduces digital tools only slowly. While this is often explained by the inertia of bureaucracy, we assert that also elements of restlessness can be a problem. We observe that at least three sources of restlessness play an important role in the studied organization:

- Firstly, the ongoing digitalization of modern society confronts organizations with increasing pressures to meet ubiquitous demands for continuous innovation. This “innovation imperative” (Jordan, 2014; Schulz-Schaeffer and Egbert, 2023) serves as an important leitmotif for organizational decision-making in all sectors of modern society and is a central driver for digitalization endeavors. As such, modern societies favor novelty, but their innovation imperative makes today’s organizations inherently restless. This seems not only true for economic organizations, but also for all other organizations, including political administrations.
- Secondly, in democratic political systems the dynamic of change for administrative organizations with politically determined leadership roles is exacerbated by general political processes. In these organizations, the generalized innovation imperative adds to another form of external restlessness that arises from the inherent dynamics of democracies, where changes of government through elections lead to corresponding changes in administrative structures and leading personnel (King and Thornhill, 2003; Luhmann, 2010).
- Lastly, organizational principles such as the “rotation of personnel” (Böhret et al., 2006) are another source of, in this case endogenous, restlessness in modern bureaucracies. This principle is laid down in various state laws and in the regulations of several administrations. In Germany, especially in governmental administrations, public officials at the middle management level regularly have to switch positions and departments. But constantly changing personnel might lead to switching middle-term-goals and a loss of knowledge, thus increasing organizational restlessness.

While traditionally the ideal type of modern bureaucratic organization in the sense of Weber (1972) is understood as a prime example of organizations that are based on stability and routine, the three described dynamics make it difficult to ensure enduring structures, which are necessary conditions to fulfill an encompassing digital transformation. The central problem is that all three aspects imply change, but they foster different forms of change that are not coordinated with each other and with the request to digitalize. Thus, combined with other sources of restlessness today’s innovation imperative seems to contradict the traditional modus operandi of bureaucracies. Conceptually, we will show how external and internal pressures to change can endanger the balance between variety and redundancy that every organization needs to operate properly (Luhmann, 1988, 2018; Esposito, 2021) and define a disbalance in favor of variety: organizational restlessness. Our focus on political bureaucracies will enrich not only the understanding of the risks of organizational change, but also the potentials and consequences of the organizational handling of the digital transformation. Thus, our case study contributes to the enhanced understanding of the organizational dimension of the ongoing digitalization of society (*cf.* Kette and Tacke, 2021; Manhart and Wendt, 2021). Our case study illustrates how disruption, variety and continuous change can in fact be problematic for organizations and that, contrary to popular belief, continuity, redundancy and stability are necessary conditions for the sustainable organization of the digital transformation of society.

Our contribution begins with a review of existing research on organizational and administrative change along with contributions on problems and risks of continuous change. Following the literature review, we outline our conceptual framework by discussing the distinction between variety and redundancy as developed in Niklas Luhmann’s organizational sociology (Luhmann, 2018) (2). After brief methodological remarks (3) we use the case study of the Federal Ministry of Defence to show how digital transformation is accompanied by a high degree of organizational restlessness. We empirically illustrate the aforementioned three forms of restlessness and show how the Ministry is characterized by the general societal innovation imperative, regular leadership changes and staff rotations, which lead to different and uncoordinated forms of change (4) we then discuss our findings in the light of the relationship between diversity and redundancy in organizations (5). In conclusion (6), we summarize our findings and consider options for future research and the management of innovation in administrative organizations.

## 2 Organizations between stability and change?

In a turbulent (Ansell et al., 2017), crisis-prone (Holton, 1987; Stewart, 2013) and complex (Luhmann, 2012) society, organizational change is considered essential. Modern organizations are expected to adapt to societal crises (Nonaka and von Krogh, 2009; North et al., 2018), to continuously learn (Gherardi, 2001; Argote, 2013; Örtenblad, 2020) and to build structures that enable them to rapidly cope with new and unforeseen challenges (Moreira, 2017; Gümusay et al., 2022; Zaccaro et al., 2023).

Organizational change is generally considered highly desirable and is discussed in several heterogeneous and yet interlinked discourses in organizational studies and sociology. At least four variants of conceptualizing organizational change can be differentiated. First, many works on *organizational knowledge management* address organizations’ ability to change as a necessary feature in modern society (*cf.* Nonaka and Takeuchi, 1995; Nonaka and von Krogh, 2009; Dalkir, 2011). The vast literature on knowledge management asserts that modern organizations must constantly create new and supposedly better knowledge to cope with the challenges of the complex and turbulent modern society. Constantly optimized organizational knowledge and its management, it is argued, allows innovation and the better handling of new problems. Especially for digital transformation processes, knowledge management is seen as necessary (*cf.* North et al., 2018).

Accordingly, the concept of *organizational learning* is discussed in several areas of organization research from sociology to economics, as well as in management theory (*cf.* Argyris and Schön, 1978; Levitt and March, 1988; Gherardi, 1999, 2001; Argote, 2013; Gherardi and Strati, 2013; Örtenblad, 2018, 2020). Beyond individual learning practices, it is asked how organizational learning capacities that are (relatively) independent of the knowledge of single employees can be strengthened and how individual and organizational learning processes can be combined constructively (Senge, 1990; Quinn, 1992). The “intelligent” organization, it is argued, constantly considers and improves its structures in order to effectively use and combine individual and organizational learning to enhance flexibility. Accordingly, the recent discussion on “agile” organizations (Moreira, 2017; Zaccaro et al., 2023) in different disciplines focuses on the optimization of organizational action, its flexibility and its ability to change.

A third discourse on the supposedly positive effects of organizational change centers on the role of *organizations in innovation processes* (Cohen and Levinthal, 1990; Tushman and Rosenkopf, 1992; Powell et al., 1996; Edquist and Johnson, 1997; Hage, 1999; Hasse, 2003; Besio and Jungmann, 2014). Organizations are seen as necessary in order to develop and stabilize innovations; vice versa, innovations are seen as a vital way for organizations to stay on top of their game, hold or improve market positions and/or improve organizational processes. Continuous innovation by and in organizations is also seen as a necessary and positive ability, while the problematic aspects of permanent change often remain quite obscure.

The bulk of the contributions on knowledge management, organizational learning and innovation ask how organizations can be more flexible when facing today’s challenges. The ability to continuously change is seen as an indispensable feature for modern organizations in order to survive in a societal environment in permanent flux. That such demands for comprehensive flexibility can in fact cause problematic consequences, is not addressed with the same intensity. In contrast, our contribution focuses precisely on problems caused by continuous organizational change and uncoordinated change.

Here, we connect to some available contributions which stress the dark side of organizational change (*cf.* Hannan and Freeman, 1984; Levinthal and March, 1993). It has been shown that organizations typically resist fundamental changes (March, 1991; Xiao, 2021); organizational learning can be a problem when it is too fast (Levitt and March, 1988); learning at a lower level may hinder more important learning at a higher level (Levinthal and March, 1993); an exaggerated tendency to innovate can jeopardize long-term improvements (Levinthal and March, 1993; Pronzini et al., 2012); and demands for continuous innovation can cause a too rapid adaptation based on limited experience (Hannan and Freeman, 1984; Hasselbladh and Ydén, 2019). These studies illustrate several risks of change and learning, while problems of continuous organizational change are discussed more prominently in studies of *organizational reforms* (*cf.* Aberbach and Christensen, 2014). The study of (often unsuccessful) reforms highlights the risks of organizational change. Too many reforms (and reforms of previous reforms) may lead to a loss of orientation and promote structural inertia: when everything changes all the time, paradoxically everything seems to stay the same. As Brunsson (2009, p. 94) points out, continually reforming organizations “may also inhibit opportunities for manipulating the organization in a specific direction: “changefulness” is not the same as “changeability.” On the contrary: changes in certain regards – which may improve organizational flexibility, agility and adaptability – can make the organization actually more change-averse in other aspects (2009, p. 91). Trying to enhance flexibility can lead to structural inertia. Thus, change and stability, continuity and disruption appear as two sides of the same coin and organizations must balance both sides adequately to combine flexible and stable structures.

To conceptualize that every organization needs to balance stability and change, we use Niklas Luhmann’s distinction of variety and redundancy (Luhmann, 1988, 2018; Esposito, 2021). Luhmann conceptualizes organizations as social systems which operate via the (re-)production and linking of decisions. Current decisions allow for the further production of consecutive decisions and connect to former decisions. This process of reproduction of operations is called autopoiesis. Organizations are thus autopoetic social systems based on decisions. In this process, decision premises (March and Simon, 1958) develop as structures which facilitate the linking of current decisions with former decisions. Luhmann names three forms of decision premises: (1) decision programs that define the conditions for the factual correctness of decisions. Such programs can be differentiated in conditional programs which define procedures in an “if-then” schematic and purposive programs which set specific purposes but leave the means to achieve them relatively open. (2) The second type of organizational structure is communication channels, which describe the relations of different organizational positions. (3) The third form of decision premises in organizations is personnel; Luhmann emphasizes how it makes a difference which person occupies an organizational position: Person A might decide differently than person B (Luhmann, 2018).

For Luhmann, to keep operating, organizations as systems are in permanent oscillation between variety and redundancy regarding their specific (internal and external) environments. Redundancy describes how organizational decisions constrain external contingencies and ensure internal stability over time via appropriate structures. While this may enhance internal consistency, a high level of organizational redundancy also leads to a reduced capability to take complex environments into consideration. On the other hand, organizational variety describes an organization’s ability to adapt to heterogeneous circumstances. Organizational learning capabilities and the ability to flexibly change structures should ensure that organizations can adjust to dynamic environments. In the perspective of Luhmann, both redundancy and variety are relevant for the survival of organizations as systems operating in a complex environment.

We describe the situation of an imbalance in favor of variety as “restlessness.” We connect to the description of Hunt (1972) of organizations as inherently restless social systems. As they must continuously adapt to ever-changing contexts, organizations are constitutively restless. For Hunt, restlessness is a positive trait of organizations: he argues that they have to strive for a certain level of restlessness to ensure their “requisite variety” (Ashby, 1956), i.e., their adequate level of internal complexity equalling the complexity of their environments. But restlessness in certain aspects might be dysfunctional for organizations and even lead to structural inertia. We use the term “restlessness” to point out these problems.

We aim to show that restlessness can even characterize bureaucracies. The ideal type of modern bureaucracy, as Weber (1972) has shown, is normally seen as a prime example of an organization with a high degree of redundancy, while economic, market-oriented organizations are supposed to have a high degree of variety (*cf.* Courpasson and Reed, 2004; Olsen, 2008). Starting from these considerations, research is endeavoring to show under what circumstances bureaucracies can achieve flexibility and how they should be managed to become more flexible (e.g., Bigley and Roberts, 2001; Briscoe, 2007; Lazega, 2020). By describing the “restlessness” of bureaucratic organizations, we show that also public administrative organizations, normally seen as inert, change-averse and redundant in their internal structures, can in fact be confronted with different forms of variety. In particular, we consider that (1) the omnipresent innovation imperative in society, (2) the variation in leadership as a result of democratic processes and (3) the rotation of personnel show that bureaucracies are confronted with various sources of change which can create an imbalance between variety and redundancy. (1) The modern innovation imperative (Jordan, 2014) transforms innovation into a societal value (Godin, 2015) which is generally seen as positive: organizations that want to succeed are expected to innovate. However, this can lead to organizational and management behavior which strives for innovation as an end in itself without reflecting possible side-effects. (2) Change in the top positions of political organizations (Riggs, 1963; King and Thornhill, 2003; Luhmann, 2010) is inherent to democratic systems and is expected to ensure organizational adaptation to changing political interests, needs and priorities, but it can be too rapid to implement long-lasting changes. (3) Finally, personnel rotation (Böhret et al., 2006; OECD, 2016) is considered a way to renew organizations and avoid structural stiffening, but it can imply a loss of competencies and dedication. Taking these different aspects into consideration, our case study will not only show that restlessness can have different sources, but also that those heterogeneous sources of variety are often not coordinated and how this makes it difficult to deal with desirable innovations, e.g., concerning digitalization.

We define digitalization as a process in which social systems like formal organizations incorporate digital technologies like hardware, software, AI and Big Data technologies into their intrinsic processes (Lupton, 2015). With their codes, memories, and algorithms, digital tools process information in new ways and deliver outputs that may surprise other actors (Baecker, 2016, p. 18). In consequence, digital technologies have the potential to fundamentally change organizational decision-making processes and re-configure how organizations deal with their external environments (Baecker, 2021). Moreover, systems-theoretical conceptualizations of digitalization emphasize that the digitalization process and its outcomes must be understood as depending on the social context in which digitalization occurs. Regarding organizations, Büchner (2018) shows that processes of digitalization are constitutively shaped by organizational structures and processes. The specific organizational aspect that we focus on in this contribution is organizational restlessness.

## 3 Methods

Our case study is a big German public administrative organization: the Federal Ministry of Defence (Bundesministerium der Verteidigung; BMVg). Public opinion sees this organization as slow and incapable of adapting to new environmental requests (Arnold, 2024). The lengthy processes are not only repeatedly criticized as regards the maintenance and procurement of materials, but also concerning the introduction of digital technologies (e.g., digital radios). Even if the organization recognizes these problems (Rieks, 2023, p. 108), few improvements have been made to date. Concerning digitalization, this problem does not only affect the studied big ministerial bureaucracy; the whole public sector in Germany introduces digital devices very slowly (Klenk et al., 2020). Rigid bureaucratic structures, old procedures and standards, as well as processes for storing and circulating knowledge, are considered central obstacles. We add to this explanation the issue of restlessness.

We use results from the project «Leadership Cultures in the Digital Age. The Case of the Bundeswehr’». This project focuses on processes of digitalization in public administrative and military organizations and the obstacles to rapid digitalization. The goals and expectations related to the organization’s digitalization were first worked out from several official documents on digital transformation and strategy papers, as well as reports on the current state of digitalization. The questions for the expert interviews were developed on this basis. We conducted 20 expert interviews with members who shaped and were responsible for the digital transformation of the organization. We also conducted 14 topic-centered interviews and various participant observations of workshops and meetings in three selected areas in which digital tools for analyzing large amounts of data are used or are in development. We supplemented these interviews with informal conversations on the sidelines of the meetings. For the systematic structuring and evaluation of both the interviews and other types of data, we performed a qualitative content analysis (Mayring, 2014), allowing simultaneously for a theory-oriented approach and systematic deductive-inductive category formation. First we coded the following main categories: forms of digitalization, challenges of digitalization, perceived advantages and risks, as well as obstacles to digitalization. Focusing on the obstacles to digitalization in detail, we found out that on the one hand classical bureaucratic problems such as complicated processes for authorizing and financing projects, hinder digitalization (as we theoretically expected), but on the other hand interviewees often stressed that other aspects related to continuous, rapid and uncoordinated change also make this process difficult. We thus coded these aspects systematically under the concept of “restlessness” which we theoretically developed based on the definition of Hunt (1972) and a system-theoretical understanding of organizations (Luhmann, 2018). Moreover, in the coding process we inductively identified three main sources of restlessness that hinder the digitalization process in the studied organization: the modern innovation imperative, changing political leadership and the rotation of personnel. A literature search has confirmed that these phenomena are well known, but their problematic consequences are understudied.

## 4 Sources of restlessness in a large administrative organization

### 4.1 The modern innovation imperative

Beyond the traditional economic and technological paradigm (Rammert et al., 2018), nowadays innovation is a desired and desirable goal of virtually every sector in society. Modern society favors novelty, innovation becomes a “value *per se*” (Godin, 2015, p. 8), a modern semantic that describes change as inherently positive and a chance to solve problems better than in the past. Innovation is inherently associated with positive change and, as such, is a central source of organizational change. In a nutshell: today’s society is characterized by a general innovation imperative (Jordan, 2014; Schulz-Schaeffer and Egbert, 2023). Thus, even political organizations and bureaucracies, seldom seen as particularly innovative, are under pressure to optimize their actions with innovative technologies, innovate political concepts, and introduce novel solutions to current problems. Governance itself must be innovated (Voß, 2018) and political-administrative organizations are the places in which and the actors by whom this must be realized (*cf.* Jöstingmeier, 2023). The innovation imperative can thus become a source of continuous restlessness: (political) organizations always try to innovate with the expectation of optimizing their actions and decisions.

The innovation imperative is related to digitalization in the sense that digital innovation is considered highly valuable and generally associated with improvements, while the difficulties in digitalization processes for political organizations are often kept latent. This positive relationship between digitalization and innovation can be found in the strategy papers of the studied organization (BMVg, 2019a,b, 2022, 2023). With the digitalization process, the organization aims to optimize and modernize defense and military activities as well as administrative tasks (BMVg, 2019a, p. 14, 15). In this context, innovation is crucial and several organizational units and structures have been developed to foster innovation (e.g., Cyber Innovation Hub, Founders UniBw, etc.) (BMVg, 2019b, p. 1).

Accordingly, in our interviews employees formulate the need and the wish that the organization modernize itself and embrace the rapid innovation process concerning digitalization which is currently unfolding at the level of society. In our empirical material, we could see that advancing digitalization in the organization is desired and required, and that keeping pace with society is crucial. As the following short statement illustrates, often the need to follow digital development is formulated, while at the same time the fact that the organization is not yet capable of innovating at the required pace is noted:

“The portfolio and the possibilities and the innovation steps change every 2 years, and we have not yet found a way to keep up” (El10, p. 2 L. 75–77).

This effort to embrace innovation, along with the critical acknowledgment that the organization digitalizes only slowly and has “an implementation problem” (EI4 94–95), can have positive effects on the process of digitalization. This organizational attitude contributes to developing an open and progressive organizational culture. However, it can also lead to the belief that every digital innovation is positive *per se*. As a consequence, new tools are introduced only because they are new, without an analysis of the necessity, practicability and possible unintended consequences of their use. We describe an example of such experiences:

“I: There is this [Messenger]. Do you use something like that, for example?

E: No. […] There was a [Messenger] thing here for the first time, which also came from the [innovation unit], I think. […].

I: Why is that not practical for you?

E: Because I can see my men. When I’m on [the vehicle], I must turn off my cell phone, otherwise the radio is jammed. And we do everything that’s important over the phone. And everything that is unimportant and coordinative via WhatsApp. At least everyone has that. And that’s always the case with them, no matter what they install on it, someone always does not have it. The only thing everyone has is WhatsApp.

I: I can see there’s still a lot to do.

E: It’s information like “Collect for sports at 11:30” or something like that. You do not need a super-encrypted super system for that.” (TI5 l, l. 535–556).

From the perspective of some interviewees, the desire to promote digital innovations also leads to counterproductive developments and a loss of established capabilities which are considered valuable. Such competencies would also allow the organization to function in cases of the collapse of digital infrastructures and are addressed under the concept of resilience. In the official documents, resilience is a central aim and digitalization is considered a relevant means to increase it (BMVg, 2022). Our interviewees stick similarly to the necessity to be resilient and recognize that digitalization, when it includes redundant structures, can increase resilience (EI7, EI21). However, they also stress that the loss of competencies related to digitalization can on the contrary reduce resilience. For our interviewees, redundancy of competencies guarantees viable alternatives for possible failures in some parts of the organizational technical infrastructure:

“The possibility for the commander to [directly observe environmental data] and see what this looks like in 3D with my own eyes no longer exists. In other words, they look out via monitors via cameras. Based on the view via the monitors, they get a picture of the situation. The possibility of leading via hand signals and saying: “Here without us having to talk, I will point in this direction and then you drive there,” no longer exists at all […] we are losing that. By digitizing certain things at the lowest tactical level in the sense that we no longer need personal contact, computers do it all for us. And that is a danger that we are currently indulging in a little bit” (EI2, l. 659–668).

The affirmative attitude toward novelty also has the consequence that the first phase of the innovation process, which includes the generation of new ideas and the invention and development of new products, is emphasized. The implementation of new devices is less important than the development of additional new ones. So, a lot of projects are started and prototypes developed, presented and discussed, but they are not implemented and integrated on a large scale in day-to-day activities. This can be described as “hypertrophy” of innovation: an exaggerated tendency to innovate, which hinders the accumulation of skills and long-term improvements and can thus ultimately become problematic (concerning the risks of innovation: Hannan and Freeman, 1984; Levinthal and March, 1993; Pronzini et al., 2012). There is a common phrase used not only in the organization we analyzed, but also in the public debate to refer to this situation: “Tal des Todes” (Valley of death) (Die Bundesregierung, 2023), a standstill which can befall a new device after being successfully developed.

Thus, the most perceived problem of digital transformation is the slow implementation of new devices (EI4 94–95). Since the stress on implementation including schooling people to use digital tools in their day-to-day activities is relatively low, often the introduction of new devices in everyday work stagnates. As a consequence, employees are more and more skeptical about the digital transformation:

“It takes us quite some time to get certain tools up and running. For example, as we have just noticed together, how difficult it is to get together in such an electronic conference on our systems and that frustrates people and very quickly leads to a defensive attitude” (EI8, l. 55–58).

While flagship projects are being driven forward because they show the ability of the organization to innovate, there is a lack of basic elements of digitization in day-to-day work. The introduction of such “boring” elements of digitalization proceeds only slowly, but, as our interviewees stress, such elements are important and even an indispensable precondition to increase efficiency and be capable of digitalizing in more intriguing dimensions. In the following statement, the astonishment related to the lack of such simple elements is expressed in the form of rhetorical questions:

“Why is it so difficult to get WLAN in properties where young members of the armed forces are being trained, for example? Why did it take us ages and 3 days to get a system like WhatsApp Messenger up and running? Yes, but these are IT issues that always overlap with what digitalization is supposed to achieve” (Interview El8, l. 60–62).

### 4.2 Changing political leadership

A second form of external restlessness stems from the inherent dynamics of democracies, in which governmental changes through elections cause corresponding changes in administrative structures and leading personnel (*cf.* Luhmann, 2010). In several public organizations, leadership positions are not filled autonomously, but personnel is recruited externally (Riggs, 1963; King and Thornhill, 2003, p. 123 f.), i.e., by (party) politicians, and changes in the light of election results. As such, democratic elections are a source of restlessness because they have the potential to cause changes in preferences, goals and normative values, depending on the political stance and interests of the current role-holder. While bureaucratic structures fundamentally serve as a protective mechanism against the “hijacking” of public administrative organizations by political parties (Perrow, 2014), it nevertheless makes a difference which politicians lead public administrations. The change in political leaders with different political positions influences goals, policies and the prioritization of tasks. Of course, political change does not have to occur at every election, but in the case of the observed organization, this was the case. Additionally, relevant top positions have also been changed during one period of legislation.

Political leaders often have “visions.” These are important for the external visibility and profiling of politicians and political organizations, and are an important contribution toward their legitimation. However, visions are considered dangerous internally because people with strong ideals fight to change present conditions and this binds economic resources and personnel that could be better used elsewhere (conversation 14,022,024). Particularly difficult are visions that imply structural reforms to optimize work processes. The organization under study has gone through several reforms and reorganizations of this kind over the last decades. Even if these attempts set different foci, one main aim was reducing overarching bureaucracy (Schelleis, 2012, p. 153). Often mentioned is one attempt to introduce and increase the relevance of target agreement and quantitative performance indicators (EI1 l. 391–394). This reform attempt also emphasizes digitalization as a means of increasing performance. Due to the similarity of its objectives with some characteristics of the private sector, this reform has often been criticized, but an additional criticism is that this reform has not yet been completely implemented and has therefore caused incomplete change:

“Under-state Secretary [X], with her [consulting firm] past, naturally brought a completely different world of thought into this and tried to implement a lot in this mindset, I’ll put it this way, but at the end of the day she left too soon and underestimated how long it would take for this to really become anchored here” (EI 1, l. 470–473).

The subsequent leadership places less emphasis on this endeavor (including the effort to digitalize) so one cannot expect a full implementation of this reform in a few years. At the same time, old, reliable work practices have been unsettled by this reform attempt.

With particular regard to digitalization, several interviewees assert that it is important that the top of the organization is willing to digitalize and that this willingness has continuity and does not change with every change in leadership:

“But what is needed from my point of view and why we are not any further forward is because we have no continuity at the top of the organization, in the sense that the leadership wants exactly such a thing” (EI 1, l. 473–474).

The lack or presence of the political will to digitalize is a central issue for the studied organization, because, as formulated in the following quote, in this organization political decisions shape the entire organization and are more relevant than factual or technical considerations:

“The political guidelines are above everything, in all areas. You can say 10 times “Yes, but technically there are arguments for and against it. Or should not be in this area.” If the responsible minister or under-state secretary says “This is what we are doing, this is how it should be implemented,” then this is how it should be implemented.” (TI12, l. 526–530).

Due to strong hierarchical structures, the influence of the top of the organization and its attitude toward digitalization is encompassing. Above all, it is the hierarchical assessment system that influences the willingness of middle management to digitalize:

“This awareness at the top naturally leaves its mark. The management levels below are generally already very career-oriented and this also shapes the management levels below, because they ask themselves: what do I have to do to ultimately be evaluated well by the levels above, and if this question of let us say goal achievement is less relevant than the fact that you get through your period of service as error-free as possible, then of course this does not support the actions of the managers” (EI 1, l. 484–489).

When the political willingness to digitalize changes, discontinuity in personnel at the higher level is mirrored in a discontinuity in priorities, concrete work activities and the use of digital technologies. Pointing to the example of a specific data analytics tool, one interviewee stresses this discontinuity:

“If you look at what the old minister and [under-state secretary], who also played a key role in tinkering with the [tool X], wanted back then and what the current minister or the current under-state secretaries [want], the approaches are completely different.” (TI12, l. 460–464).

Since this tool has no priority for the succeeding political leadership, there is no answer to the concrete problems formulated by operative units and the implementation of the tool is slowed down (meeting 12,062,023).

The changing organizational top also implies the problem that the phenomenon of shifting responsibility is exacerbated (*cf.* Brunsson et al., 2022). In the view of our interviewees, it is difficult for politicians to take responsibility for mistakes and there is a tendency to shifting responsibility to others (EI3 l. 816–820). Rapid changes of leadership also enables responsibility to be shifted to the former leaders and along with blame for mistakes or a too-slow process of digitalization.

### 4.3 Rotation of personnel

The third source of restlessness for the studied organization is the principle of the “rotation of personnel” (Böhret et al., 2006). This source is endogenous, since it depends on the organizational rule that every defined number of years managers get new positions in different units. In Germany, the rotation requirement, the implementation of which was decided in 2008 by the German Bundestag, is central to public administration. This requirement is regulated by state law and specific regulations of single public organizations. Public officials have to switch positions and departments at regular intervals. The rationale underlying this procedure is to combat corruption, avoid long-term narrow contact with employees and clients, maintain personnel that can be flexibly used in different tasks, but also to develop individual competencies and increase creativity (*cf.* OECD, 2016).

Our interviewees also recognize the positive aspects of the rotation of personnel: it is well known, they argue, that people who stay for too long in the same position tend to take over their turf, increase their own discretionary boundaries and develop exaggerated self-confidence:

“[X] considers himself the little god and the focus is no longer so much on the leadership task, but rather on self-realization. […] In the past […] we also had [managers] who were in their posts for a surprisingly long time. It was difficult to get information into them because when they do something like that for a few years, they always have to resist the temptation to know everything, to become the ultimate expert.” (EI6, l. 1,005–1,008).

For individuals too, it could be advantageous to change positions because different competencies can be learned in different units: one can work in more operative or strategic units, with different spans of control, in central or peripheral units, and so on (conversation 14,022,024). However, this supposes that the individuals are willing and able to continuously learn the specificities of the new units. This is considered unrealistic and happens only under exceptional circumstances:

“We often lack […] knowledge management for the subsequent post: we do not take the time to take it with us, […] we lack change management, or we simply do not have the time to familiarize ourselves intensively with the existing things so that we can then apply them” (TI1, 507–511).

More often than the advantages, the problematic restlessness caused by the rotation principle comes up in our interviews and during the meetings we attended. Normally, middle-management personnel rotate every 3 years and if we add normal fluctuation due to illness, maternity leaves, pensions and so on it becomes very difficult to complete specific projects (EI3 l. 519–524). Especially in governmental administrations with, as we have seen, a changing organizational top, additional constantly changing personnel positions at the level of middle management might lead to fast-shifting goals. Typically, people begin projects when they change positions, but often these projects cannot be completed in the short period of time available. Projects are initiated, but not completed, prototypes developed, but not implemented, ideas developed and discussed, but not concretized. Considering that a person needs some months to get acquainted with new units and that once a project is formulated a long period (at least 1 year) is needed to get funding and prove the juridical correctness of a project (for digital projects in particular) (meeting 06122023), it is not realistic to think that a project could be developed and completed in 3 years. As a consequence of the rotation principle, projects must be continued by the successors. However, they are not really interested in pursuing projects of others because the incentive system pushes them to increase personal visibility and this can be at best reached with innovative and brilliant projects of their own. With this in mind, organizational members recognize that it is important to ensure that people stay in a position for a sufficient amount of time so that they can conclude their own projects and have no incentive to procrastinate tasks by waiting for the next position. An appropriate duration of the position would imply that.

“[employees] cannot hide, somehow do nothing and when they have been there 3 or 4 years, they can have a say and touch things and, above all, finish things” (EI6 l. 1,016–1,017).

The rotation of personnel also leads to a loss of knowledge. This is particularly important in knowledge-intensive units and in every project that is concerned with complex technologies, especially digital technologies. This problem is formulated in the following example for a digital tool that is used by 2000–2,600 organizational members who enter their ratings, comment on ratings of others and/or summarize them at a higher hierarchical level. Since the personnel who operate these data entries change approx. every 3 years, the need for training is high:

“That’s a bunch of people who of course regularly change their posts and have to familiarize themselves with this tool again and again.” (TI2, l. 425–427).

The problem is also exacerbated because people do not work regularly with this tool, simply because there is no urgent need to make a data entry every day, but also because the tool is considered by many to be a waste of time. For this digital tool, human analysis (military judgment) and data entry is needed. In this case, the acceptance of the tool is an important condition for avoiding sloppy data entries (meeting 05062023). For this reason, it is important for users to understand the objectives, capabilities and limitations of the tool. They should be able to answer the question: why should I use my valuable time trying to evaluate my activities and enter my comments in a program? The loss of meaning associated with specific tools caused by the rotation of personnel is even more problematic than the loss of practical knowledge as to how to use them. As a consequence, knowledge management and continuous discussion become crucial, as this statement stresses:

“My predecessors, who really traveled around the country and tried to explain why it is important to do this and the staff changes […] and the idea of why we are actually doing this and who actually benefits from it, I do not think it has been passed on, so it has not been inherited, so that many requests come in today” (EI2, l. 302–304).

We could experience a similar loss of knowledge and meaning in a meeting where the completely changed staff after 6 months in charge were not yet acquainted with the technical and organizational details of a big data analytics project that concerns their activities. While they were capable of discussing general questions, it was very difficult to discuss specific functions, limits and improvements to the tool (meeting 12,062,023).

The chance to stay in a position, if one wishes, can be granted under specific circumstances, but this can endanger careers, as one very competent civil servant told us: he decided to stay and to fight for a specific project. Over the years, he built alliances as well as internal and external contacts, he wrote documents to analyze the technical and social impact of the introduction of a specific tool and also managed to convince skeptics of the tool’s effectiveness. At the time of our encounter, the tool was a well-developed prototype, was in use in a few units and would have needed more support to be introduced on a broader scale. However, our interlocutor was advised to change position and to abandon this project for the sake of his (already endangered) career (conversation 120,623).

## 5 Discussion

In the analyzed organization, there is an imbalance between redundancy and variety in the time dimension. Since this imbalance favors variety over redundancy, this situation can be described as organizational restlessness. We could observe three sources of restlessness: the innovation imperative of modern society as a form of semantic restlessness, the exogenous restlessness caused by the regular exchange of leadership in light of democratic elections and finally the endogenous restlessness based on the bureaucratic principle of the rotation of personnel. These sources of restlessness affect the organization not only singularly, but also in combination.

Modern society’s generalized innovation imperative (Jordan, 2014; Schulz-Schaeffer and Egbert, 2023) is the first source of exogenous variety. This imperative is not economic or political coercion, but a semantic construction that discursively circulates in the public sphere. The semantically stressed positivity of innovation causes a permanent restlessness as variety, i.e., innovation, is seen as worthwhile in itself and could therefore be introduced without proper planning. So several innovation projects are initiated without reflecting the concrete organizational consequences and the exact needs of the personnel. As a consequence, numerous difficulties arise later in the day-to-day work and this even causes a defensive attitude in the employees which makes it difficult to sustainably stabilize digital innovations. The continuous attempt to change induced by the innovation imperative ultimately leads to an imbalance favoring variety.

The democratic principle of replacing leadership after elections is exogenous to administrative organizations and characterizes the political systems in democracies (King and Thornhill, 2003). Even if this principle does not arise from organizational, but from political needs, it profoundly affects the organization studied since it leads to a permanent restlessness at the top of the organization. It is not uncommon for a former minister of Party A to want to pursue certain projects and innovation endeavors, while the new minister of the winning Party B might be completely uninterested in their predecessor’s projects. Their under-secretaries of state (Staatssekretäre) do not ensure administrative continuity, but implement the political line of the relevant minister: the new minister and under-state secretary might have completely different plans and, as a result, shut down old projects and promote other approaches. Here, this form of exogenous restlessness amplifies the problems caused by the innovation imperative: implementing digital innovations often does not fit into the relatively tight schedule of democratic election cycles; implementation may need more time than one minister’s legislative term. As a consequence, it is politically advisable to start new, highly visible digitalization projects in every legislature instead of sticking to projects introduced by others. Changes in leadership, then, are a source of variety that works as a hindrance for profound transformation processes.

Finally, the rotation of personnel at the level of middle management sanctioned in law and regulations is an important source of endogenous restlessness. Here, variety is constantly (re-)introduced in the organization, not only at the top, but also by changes of personnel at the operational level, making it more difficult to guarantee continuity, thus increasing decision uncertainty and instability from within. While the rotation principle has the advantage of counteracting personal power concentrations, the rotation of personnel is at the same time a source of problematic variety. A high rate of personnel rotation leads to a loss of established knowledge necessary to adequately stabilize innovations. With different personnel, different goals are emphasized and projects that had started beforehand might be ignored or at least not be followed through as might be necessary. In the end, this again might lead to a standstill in the implementation of innovation projects. Added to the politically motivated change at the top, personnel rotation at the level of middle-management puts the organization in a situation of too much simultaneous uncoordinated change and undermines the necessary continuity of leading personnel at least at some hierarchical levels. Considering that continuity in leadership is often essential to stabilize even limited innovation (Rehman et al., 2024), the difficulties that restlessness causes for fundamental changes become evident.

Considering all three sources of restlessness together, it becomes obvious how different and, more importantly, largely uncoordinated forms of variety can impede sustainable innovation. “Changefulness” in the sense of Brunsson (2009, p. 94) hampers “changeability” and different sources of change might in fact lead to stasis. As our observations show, it is not (only), as often assumed, the bureaucratic inertia of the organization that hinders digital innovation; in fact, it is also an uncoordinated variety that makes it difficult to think digitalization projects through and stick to them for the time necessary to integrate them properly into organizational processes.

Again, stagnating digitalization is not (only) caused by the staleness of bureaucratic structures, i.e., structural redundancy, but by the opposite: too much variety. Changefulness regarding personnel and projects impedes the changeability of digital innovation; heterogeneous and uncoordinated variety contributes to the slowing down of the highly desirable digital transformation. Formulated in the concepts of Niklas Luhmann: not only too much redundancy can constrain variety, too much variety of some organizational traits can also hinder variety in others. In terms of decision premises, we could observe high rates of change in particular concerning the premise “personnel” at more organizational levels. Additionally, the semantic of innovation, along with the need for the political leadership to legitimate their actions, can lead to rapid changes in priorities which affect in particular purposive programs. Rapidly changing goals, priorities and projects imply that the factual dimension also does not guarantee a stable orientation. The result is that too many elements change at the same time, which threatens organizational stability (Luhmann, 2018, p. 189-190).

It is hard to characterize which kind of variety is fruitful for an organization. In addition to the imbalance of variety and redundancy, and the absence of coordination between different kinds of variety, our example shows that one can also consider that variety at one level can hinder variety at another. This aspect echoes the idea of myopia of learning that can imply that learning at a lower level may hinder more important learning at a higher level (Levinthal and March, 1993). In our case, we can observe how high variety at the level of basic structures such as leading personnel may reduce the chance to introduce variety (in our case digital innovation) at the level of decisions on digital tools. The high variety of personnel produces restlessness and does not allow a stable point of reference to develop, reflect, implement and stabilize the use of specific digital devices.

In the studied organization, an unfavorable combination of high personnel variety and old bureaucratic procedures (conditional programs) also hinders the process of digitalization. For example, numerous innovative projects fail in the implementation phase because the process of handling funding is very slow, since it involves several approval steps and revisions to be carried out by a large number of organizational units with different interests and priorities (BMVg, 2024). Because of cumbersome bureaucratic decision premises, more time is needed to get a project started. This situation makes the rotation at the middle-management level even more problematic because it is rare that a project can be seen through from start to finish by a single project leader.

## 6 Conclusion

Our analysis shows that organizational change is not always positive, but can on the contrary make it difficult to realize specific organizational aims, including highly desirable transformations such as digitalization. With this observation, we contribute to the understanding of organizational change by stressing its risks. In particular, we point out the imbalance between redundancy and variety, as well as the problem of uncoordinated sources of variety. Our observation about changes in personnel additionally supported the idea that changes at one level may hinder changes at another level: in our case, too many changes in personnel make digital transformation difficult. With our results, we contribute to an enhanced understanding of how organizations deal with the dilemma of changing and at the same time maintaining continuity. In particular, we show how constant, uncoordinated forms of variety, which we define as “restlessness,” can in fact impede methodical digital transformation. By analyzing organizational restlessness as an obstacle to digitalization, we contribute to understanding the enabling, but also limiting role of organizations for digital transformation (*cf.* Kette and Tacke, 2021; Manhart and Wendt, 2021).

Our analysis also contributes to a deeper understanding of bureaucracy in modern society. While the classic view of bureaucracy sees the slow and cumbersome formal structures of this organization type as a central problem (*cf.* Courpasson and Reed, 2004; Olsen, 2008), more recent studies show that bureaucracies too have elements of flexibility (e.g., Bigley and Roberts, 2001; Briscoe, 2007; Lazega, 2020). We also observe dynamics of change in bureaucracies, but stress that not every kind or amount of variety should be considered favorable for such organizations. In the studied organization, old bureaucratic structures and procedures hinder the modernization of the organization; they are however not the only retarding elements. In the same organization, problematic redundant structures and problematic variety coexist and negatively affect the digitalization process.

To be sure, the sources of restlessness we studied are contingent on the organization observed. Several other sources of restlessness can occur in different organizations. For example, specific forms of knowledge management, organizational cultures oriented toward learning, constant technological change, changing management fashions and so on may cause restlessness and be disadvantageous for organizational decision-making processes. Comparative analysis of different sources of restlessness in different organizations would also be necessary to better qualify which kinds of restlessness should be avoided.

More attention should also be given to compensating strategies. For our case study, it is important to consider that classic political organizations compensate for political changes in personnel at the top of the organization with stable bureaucratic staff (Perrow, 2014). This staff is aware of day-to-day processes and maintains knowledge. However, in the organization studied this compensation is reduced by the fact that many positions at the middle-management level are filled by military personnel, who rotate particularly frequently. The members of our organization reflect on such problems and think of ways to increase redundancy in personnel. In particular, the question of the appropriate frequency of rotation for different kinds of personnel with different tasks is considered crucial. Another solution approach could ask about the compatibility of bureaucratic procedures requiring large amounts of time with rapid personnel changes. In our conceptual framework, this kind of reflection can be interpreted as an effort to reestablish a balance between redundancy and variety in organizations as systems operating in a complex environment. While comments on how to cope with the problem of restlessness were only sporadic in our interviews, the awareness of the need to balance redundancy and variety, including an awareness of the risks of fast change, would be particularly relevant for management and practitioners interested in systematically improving the performance of bureaucratic organizations.

## Data Availability

The datasets presented in this article are not readily available because of legislative restrictions. Requests to access the datasets should be directed to marco.joestingmeier@hsu-hh.de.
